# Sarcopenia in Chronic Kidney Disease: A Narrative Review from Pathophysiology to Therapeutic Approaches

**DOI:** 10.3390/biomedicines13020352

**Published:** 2025-02-04

**Authors:** Chung-Ching Tsai, Ping-Chen Wang, Ted Hsiung, Yang-Hsin Fan, Jui-Teng Wu, Wei-Chih Kan, Chih-Chung Shiao

**Affiliations:** 1 Division of Orthopaedics, Department of Surgery, Camillian Saint Mary’s Hospital Luodong, No. 160, Zhongzheng S. Rd., Luodong Township, Yilan County 26546, Taiwan; tcc5068@yahoo.com.tw; 2 Department of Medical Research and Education, Camillian Saint Mary’s Hospital Luodong, No. 160, Zhongzheng S. Rd., Luodong Township, Yilan County 26546, Taiwan; pw279@outlook.com; 3 Division of General Surgery, Department of Surgery, Camillian Saint Mary’s Hospital Luodong, No. 160, Zhongzheng S. Rd., Luodong Township, Yilan County 26546, Taiwan; oldusa29@yahoo.com.tw (T.H.); b101093073@gmail.com (Y.-H.F.); smh07137@smh.org.tw (J.-T.W.); 4 Department of Nephrology, Department of Internal Medicine, Chi Mei Medical Center, No. 901, Zhonghua Rd., Yongkang Dist., Tainan City 71004, Taiwan; 5 Department of Medical Laboratory Science and Biotechnology, Chung Hwa University of Medical Technology, No. 89, Wenhua 1st St., Rende Dist., Tainan City 71703, Taiwan; 6 Division of Nephrology, Department of Internal Medicine, Camillian Saint Mary’s Hospital Luodong, No. 160, Zhongzheng S. Rd., Luodong Township, Yilan County 26546, Taiwan

**Keywords:** sarcopenia, chronic kidney disease, diagnostic criteria, pathophysiology, muscle wasting, muscle synthesis

## Abstract

Chronic kidney disease (CKD) is a progressive condition linked to sarcopenia, a syndrome characterized by loss of skeletal muscle mass and strength, affecting a quarter of CKD patients globally. Sarcopenia has multiple paths through which it can worsen morbidity and mortality as well as decrease the quality of life in CKD, including systemic inflammation, hormonal imbalances, metabolic changes, and dysbiosis of gut microbiota. There is a regional variation in the criteria set for diagnosis, with two main groups being the European Working Group on Sarcopenia in Older People and the Asian Working Group for Sarcopenia. Management regimes such as nutritional optimization, vitamin D, exercise, correction of metabolic acidosis, and modulation of gut microbiota constitute effective intervention strategies. Emerging therapeutic options include anabolic agents, myostatin inhibitors, and anti-inflammatory treatment options. Future advances such as genomics, proteomics, and personalized medicine will open up new avenues for addressing the complex pathophysiology of sarcopenia. Hence, a comprehensive multidisciplinary approach focused on the specific needs of each patient will be vital in reducing the effects of sarcopenia and improving the situation of people with CKD.

## 1. Introduction

Chronic kidney disease (CKD), a progressive condition characterized by a gradual loss of kidney function over time, affects millions of individuals worldwide [1]. As CKD advances, patients often experience a myriad of complications, including cardiovascular diseases, anemia, and bone disorders [2,3]. In CKD, the cellular protein turnover is imbalanced, with protein degradation outweighing protein synthesis, leading to a loss of protein and cell mass and impairing tissue function [4]. Besides, CKD is associated with significant reductions in lean body mass and the mass of various tissues, including skeletal muscle [4].

Sarcopenia, a condition marked by gradual and generalized skeletal muscle mass loss and strength decline, has emerged as a significant and often underrecognized consequence of CKD [5,6,7]. A recent systematic review and meta-analysis by Duarte MP et al. [8], including 42,041 patients in 140 observational studies from 25 countries, found that the global prevalence of sarcopenia was about 24.5% without significant differences among CKD stages. The prevalence of severe sarcopenia was 21.0%, which was significantly higher in dialysis patients (26.2%) than in non-dialysis CKD patients (3.0%). The sarcopenia traits in overall CKD patients were primarily low muscle strength (43.4%), followed by low physical performance (38.6%) and low muscle mass (29.1%) [8].

The loss of skeletal muscle mass is directly associated with diminished strength and indirectly associated with worse health-related quality of life (HRQoL), increased vulnerability to undesirable outcomes such as falls, fractures, loss of independence, and, ultimately, higher hospitalization rates and mortality [5,9,10]. Consistently, sarcopenia in CKD patients is clinically significant due to its association with decreased HRQoL and increased risks of morbidity and mortality. It exacerbates the already high risk of cardiovascular complications, hospitalizations, and overall mortality in CKD patients [6]. The pathophysiology of sarcopenia in CKD is multifactorial, involving a complex interplay of metabolic, hormonal, inflammatory, and nutritional factors. Recent research has also highlighted the role of gut microbiota dysbiosis in the pathogenesis of sarcopenia in CKD. Given the multifaceted nature of sarcopenia in CKD, therapeutic strategies must be comprehensive and multifactorial. Current approaches include nutritional interventions to optimize protein and energy intake, exercise interventions to improve muscle synthesis, anabolic agents such as growth hormone and insulin-like growth factor-1 (IGF-1), and anti-inflammatory treatments to mitigate the chronic inflammatory state.

Because of sarcopenia’s clinical relevance and multifaceted nature in the CKD population, a comprehensive review article providing concise and easy-to-understand knowledge from pathophysiology to therapeutic approaches for sarcopenia in CKD is critical. This review explores the pathophysiological mechanisms linking CKD and sarcopenia, discusses current and emerging therapeutic strategies, and highlights research. By enhancing our understanding of the medical community of sarcopenia in CKD, this review article aims to contribute to the field and improve the management and outcomes of this vulnerable patient population.

## 2. Methodology for Literature Search

The methodology adopted for this present narrative review gives an elaborate and rapid overview of sarcopenia in CKD, which includes pathophysiology and therapeutic approaches. We conducted a comprehensive search for studies on PubMed and Google Scholar using the following terms: “sarcopenia”, “chronic kidney disease”, “diagnostic criteria”, “pathophysiology”, “muscle wasting”, and “muscle synthesis”. Our inclusion criteria were limited to English-language research. The types of references included clinical trials, reviews, systematic reviews, and meta-analyses, with a primary focus on papers published between 2014 and 2024. Additionally, we incorporated a few references outside this range that provided valuable insights.

## 3. Diagnostic Criteria of Sarcopenia

Classification of sarcopenia varies; however, the European Working Group on Sarcopenia in Older People 2 (EWGSOP2) [11] and The Asian Working Group for Sarcopenia (AWGS) [12] are widely used (Table 1).

EWGSOP2 is an updated version of the original EWGSOP definition, incorporating scientific and clinical evidence accumulated over the past decade since their initial meeting in early 2018 [11]. EWGSOP2 provides the most widely applied diagnostic criteria for sarcopenia, emphasizing the following points: (1) Low muscle strength being a core characteristic of sarcopenia; (2) Detection of low muscle quantity and quality to confirm the diagnosis; and (3) Poor physical performance as an indicator of severe sarcopenia. Similarly, AWGS offers criteria for diagnosing and treating sarcopenia in the Asian population [12].

Several methods of evaluating muscle mass exist, though not all are widely used. computed tomography scans (CT) and magnetic resonance imaging (MRI) are considered the gold standard for diagnosing sarcopenia because they distinguish between tissues and fat. However, their use is limited by cost, accessibility, radiation exposure, and technical complexity. Dual energy X-ray absorptiometry (DXA) offers an alternative with less radiation but still faces accessibility issues. Bioelectrical impedance analysis (BIA) is more accessible and measures fat and lean body mass, but it is often used on children or teenagers due to non-standardized conditions in older adults [14]. Anthropometric measurements, such as mid-arm muscle circumference (MAMC) and subjective global assessment (SGA), also show potential for diagnosing sarcopenia and correlate with mortality in CKD patients [15].

A meta-analysis of 35 studies found that non-Asian males have a higher prevalence of sarcopenia compared to Asian males when using BIA (19% vs. 10%). However, the opposite result was observed with the DXA method (10% vs. 6%) [16]. For females, the prevalence of sarcopenia is higher than for males in both BIA and DXA methods, with non-Asian females showing a more pronounced difference (BIA: 20% vs. DXA: 11%, 10% vs. 6%) [16]. Overall, the DXA method shows a lower prevalence of sarcopenia [16].

## 4. Molecular Mechanisms and Pathophysiology for Sarcopenia in CKD

**Figure 1:**
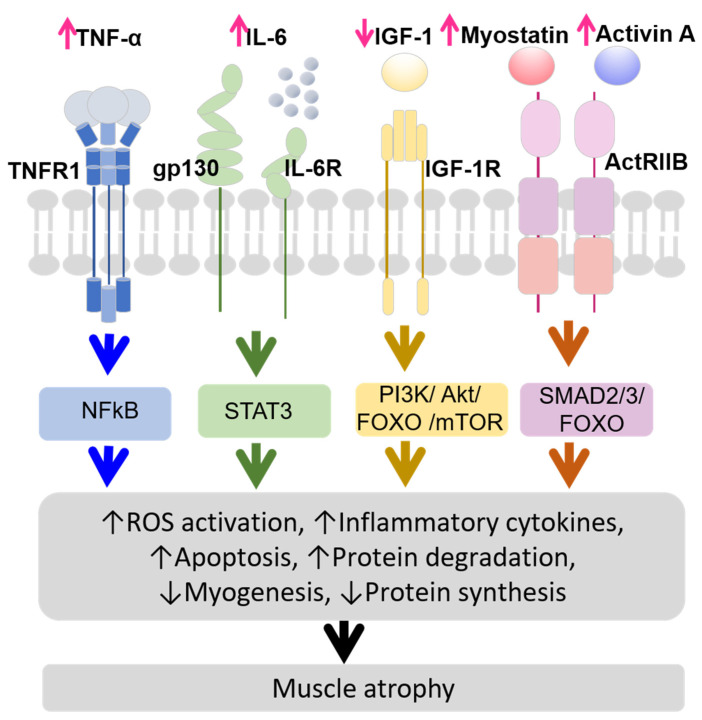
Molecular mechanisms of skeletal muscle atrophy in CKD. Abbreviations: ActRIIB, Activin receptor type IIB; FOXO, Forkhead box protein O; gp130, Glycoprotein 130; IGF-1, Insulin-like growth factor-1; IGF-1R, Insulin-like growth factor-1 receptor; IL-6, Interleukin-6; IL-6R, Interleukin-6 receptor; NFκB, Nuclear factor κB; SMAD 2/3, Mothers Against Decapentaplegic Homolog 2/3; STAT3, Signal transducer and activator of transcription 3; TNFR1, TNF type 1 receptor; TNF-α, Tumor necrosis factor-alpha.

Figure 1 illustrates the molecular mechanisms of skeletal muscle atrophy in CKD. CKD is characterized by systemic inflammation [17] and is closely associated with sarcopenia [18]. Increased inflammatory cytokines levels are linked to muscle wasting [19].

The molecular mechanisms of skeletal muscle atrophy in CKD may involve the following:

1. TNF-α (tumor necrosis factor-alpha) stimulates the ubiquitin-proteasome system [20], which is crucial for regulating the signaling pathways activated by TNF-α binding to TNF type 1 receptor (TNFR1). When TNF-α binds to TNFR1 on myofibers, it activates nuclear factor κB and reactive oxygen species (ROS) production. This activation promotes pro-inflammatory gene programs, including the secretion of interleukin-6 (IL-6) and Interleukin-1 beta (IL-1β) [21,22,23,24,25,26].
2. IL-6 levels are also elevated in CKD [27,28], and its increase or overexpression reduces muscle mass and protein metabolism [29,30,31]. IL-6 binds to glycoprotein 130 (gp130) and IL-6 receptor (IL-6R) on myofibers, activating signal transducer and activator of transcription 3 signaling and inducing suppressor of cytokine signaling 3, which inhibits IGF-1 effects, leading to protein degradation and muscle atrophy [32,33,34].
3. IGF-1 is a key growth mediator that promotes muscle health by binding to its receptor, insulin-like growth factor-1 receptor (IGF-1R), stimulating protein synthesis, and inhibiting protein degradation [35]. In CKD, IGF-1 levels decrease, impairing muscle protein synthesis and increased protein degradation. This process contributes to muscle atrophy through several pathways, including ROS activation, myogenesis, apoptosis, increased protein degradation via the PI3K/Akt/FOXO (Phosphoinositide 3-kinase/Protein Kinase B/Forkhead box protein O) pathway, and decreased protein synthesis due to disrupted PI3K/Akt/mTOR (mammalian target of rapamycin) pathway [36,37,38].
4. Myostatin and Activin A are transforming growth factor-beta (TGF-β) family members that play significant roles in muscle atrophy in CKD. Myostatin production is induced by inflammatory cytokines linking inflammation to muscle atrophy [32]. Myostatin and Activin A bind to Activin receptor type IIB (ActRIIB) and contribute to muscle atrophy by activating the mothers against decapentaplegic homolog 2/3 (SMAD2/3) and FOXO pathways, leading to increased protein degradation and inhibited muscle growth [36,39,40,41].

Overall, the interplay between inflammatory cytokines, ROS activation, impaired myogenesis, and increased apoptosis contributes to skeletal muscle atrophy in CKD. Inflammatory cytokines promote protein degradation, inhibit protein synthesis, and activate ROS, damaging muscle cells. ROS activation causes oxidative stress, leading to muscle cell damage and apoptosis, impairing myogenesis. Myogenesis reduces muscle regeneration and increases muscle wasting. Meanwhile, apoptosis leads to overall muscle mass loss (Figure 1).

**Figure 2:**
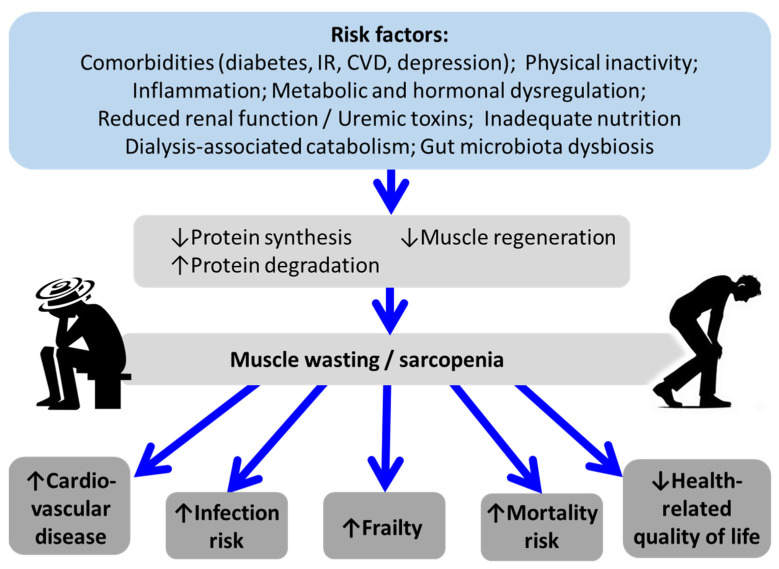
Pathophysiology of sarcopenia in CKD. Abbreviations: CVD, Cardiovascular disease; IR, Insulin resistance.

Figure 2 illustrates the pathophysiology of sarcopenia in CKD. It highlights the multifaceted impact of CKD on muscle health, showing how reduced renal function and uremic toxins contribute to muscle wasting and sarcopenia. Key risk factors include comorbidities such as diabetes mellitus, insulin resistance, cardiovascular disease, and depression, as well as low physical activity. Inflammation and metabolic and hormonal dysregulation, including hyperparathyroidism, metabolic acidosis, and hypogonadism, further exacerbate muscle degradation. Reduced renal function, uremic toxins, reduced nutrient intake, dialysis-associated catabolism, and gut microbiota dysbiosis also play significant roles. These risk factors contribute to increased protein degradation, along with decreased protein synthesis and muscle regeneration, resulting in muscle wasting and sarcopenia, which subsequently cause many consequences, such as increased cardiovascular disease, infection risk, frailty, and mortality, along with decreased HRQoL [4,42] (Figure 2).

## 5. Factors Associated with Sarcopenia in CKD

### 5.1. Inflammation

Inflammation significantly impacts CKD-associated sarcopenia. CKD patients exhibit elevated levels of inflammatory markers like C-reactive protein (CRP), IL-6, and TNF-α [42,43]. These pro-inflammatory cytokines promote muscle protein degradation and elevate levels of protein-bound uremic toxins, parathyroid hormone, angiotensin II, glucocorticoid, inflammation, and oxidative stress [44]. These alterations further inhibit muscle growth [44,45,46].

Specifically, elevated CRP levels correlate with reduced muscle strength and mass [47]. CRP exposure decreases muscle cell size and protein synthesis by affecting Akt and AMPK (AMP-activated protein kinase) pathways [48]; IL-6, through the JAK/STAT (Janus kinase/Signal transducer and activator of transcription) pathway, also contributes to muscle atrophy [49,50,51]; TNF-α activates NF-κB, leading to increased muscle protein breakdown and reduced myogenesis [52]. Besides, leptin, a hormone acting as a pro-inflammatory adipokine, can also cause sarcopenia by lowering protein intake. As an anorexigenic molecule in adipose tissue, leptin can manipulate CKD patients’ appetite, body mass, and other regulatory functions, including bone mass, immune and endocrine response, blood pressure, or sexual maturation [53]. Leptin is also associated with cardiovascular disease and arterial hypertension development, resulting from megalin-mediated metabolic degradation and a drop in normal physiologic clearance, which further leads to a pro-inflammatory uremic condition [53].

This phenomenon of inflammation often worsens along with the development of CKD [53]. During the advanced stages of the disease, the heightened production of inflammatory cytokines accelerates the breakdown of skeletal muscle [54]. Furthermore, a chronic low-grade systemic inflammatory state is common and persistent in CKD patients. Among these chronic inflammatory contributors, uremia has been identified as having a particularly profound effect on skeletal muscle [53,55]. Cytokines also regulate the synthesis of acute-phase proteins. During the advanced stages of the disease, the increased production of inflammatory cytokines can accelerate the breakdown of skeletal muscle [56].

### 5.2. Metabolic and Hormonal Dysregulation

CKD leads to several metabolic and hormonal disturbances contributing to muscle wasting and sarcopenia: metabolic acidosis increases protein breakdown through acidification-dependent ubiquitination [5,56,57]. Insulin resistance disrupts the normal insulin/IGF-1 signaling, reducing muscle protein synthesis through the PI3K/Akt/mTOR pathway. Additionally, it enhances protein degradation via the FOXO pathway, contributing to muscle wasting [44].

Accumulation of uremic toxins impairs muscle mitochondrial function and protein synthesis [5,56]. Elevated uremic toxins, such as indoxyl sulfate and p-cresyl sulfate, impair mitochondrial function and IGF-1 signaling, contributing to muscle atrophy [58,59,60,61,62]. Proteinuria is associated with the development of CKD [63]. CKD patients often suffer muscle protein mass losses induced by the inequality between synthesis and protein degradation and a drop in muscle endurance resulting from impaired mitochondrial function [44]. Similarly, the current literature also supports the stance of uremic-toxin-induced muscle loss [44].

Besides, high levels of uric acid and advanced glycation end products [64,65], thyroid hormone imbalances, and vitamin D deficiency [66] contribute to muscle dysfunction and are associated with sarcopenia in CKD patients. Furthermore, recent research found an interplay between hormones, sex, age, and sarcopenia. Inflammation derangements and hormonal factors such as growth hormone or testosterone are signs of sarcopenia-related aging [14]. Sex-specific differences are prominent in sarcopenia progression. Post-menopausal women experience a plummet in sex steroids, accelerating muscle loss, while testosterone declines more gradually in men but drops significantly after age 80, leading to sarcopenia [16].

Overall, these metabolic and hormonal disturbances create a catabolic environment, exacerbating muscle wasting and sarcopenia in CKD patients.

### 5.3. Inadequate Nutritional Status

Nutritional intake, particularly protein, is crucial in formulating and managing sarcopenia. In patients with CKD, muscle protein loss is a common consequence, leading to increased mortality risks, reduced mobility, and a lower QoL due to diminished muscle strength [67]. Skeletal muscle is the primary physiological reserve, comprising 50% of the body’s protein and 40% of its weight [68]. Research has identified that increased interleukin-8 levels, poor nutritional status, and aging contribute to muscle loss in end-stage renal disease (ESRD) patients [69]. These factors potentially lead to a decline in muscle quality and quantity [69]. ESRD patients are advised to consume more than 0.8–1.2 g protein/kg of body weight. Ensuring adequate protein ingestion is beneficial for counteracting hemodialysis-induced skeletal muscle catabolism. Additionally, muscle biopsies from CKD patients have shown reduced levels of essential amino acids, indicating decreased intracellular availability of branched-chain amino acids, which leads to protein wasting [4,70].

### 5.4. Physical Inactivity

Physical inactivity is a significant factor contributing to muscle wasting and sarcopenia by reducing protein synthesis and increasing muscle protein breakdown in CKD patients. In CKD patients, physical inactivity often results from fatigue, anemia, and reduced exercise capacity, which are prevalent in this population [71]. Additionally, uremic toxins and metabolic acidosis accumulation can impair muscle function and contribute to physical inactivity. A recent cross-sectional study by Yang et al. [72] demonstrated that physical activity independently protects against sarcopenia. Notably, lower physical activity scores were significantly linked to higher prevalence and severity of sarcopenia. It outstands the significance of promoting physical activity to mitigate sarcopenia in CKD patients [72]. The interplay between sarcopenia and physical inactivity creates a vicious cycle where physical activity decreases due to muscle loss, which in turn exacerbates muscle wasting.

### 5.5. Gut Microbiota Dysbiosis and the Metabolites

Gut microbiota dysbiosis significantly impacts CKD-associated sarcopenia. In CKD, there is a reduction in beneficial bacteria like Bifidobacterium and Lactobacillus and an increase in harmful bacteria such as Enterobacter, Klebsiella, and Escherichia [73]. This altered gut microbiota composition leads to increased production of uremic toxins like indoxyl sulfate and p-cresyl sulfate [73,74]. These toxins damage the intestinal barrier, increasing gut permeability and allowing the translocation of bacteria and endotoxins into the bloodstream [75,76]. This process exacerbates systemic inflammation, contributing to muscle wasting [76,77]. Dysbiosis also reduces beneficial short-chain fatty acid (SCFA)-producing bacteria, impairing muscle health [78]. SCFAs, such as butyrate, are crucial for maintaining muscle function and reducing inflammation [78].

Additionally, gut-derived metabolites like IS induce oxidative stress and inflammatory responses in muscle cells, further promoting sarcopenia [59]. Addressing gut microbiota dysbiosis through dietary interventions or probiotics may help mitigate muscle wasting in CKD patients [79,80]. Diets low in fiber and high in processed foods reduce beneficial SCFA-producing bacteria, impairing muscle health [78]. While protein-restricted diets can delay CKD progression, they may also contribute to sarcopenia if protein intake is too low [81]. Conversely, a balanced diet of vegetables and whole grains supports gut health and muscle function [81]. Commonly used drugs, such as antibiotics and proton pump inhibitors (PPIs), can disrupt gut microbiota, reducing beneficial bacteria like Bifidobacterium and Lactobacillus [82]. Addressing dietary patterns and minimizing the negative impact of drugs on gut microbiota can help mitigate sarcopenia in CKD patients [79,80]. Thus, diet and drugs play a critical role in the progression of sarcopenia in CKD.

### 5.6. MicroRNA

MicroRNAs (miRNAs) significantly influence CKD-associated sarcopenia by regulating gene expression in muscle maintenance and atrophy. In CKD, altered miRNA expression affects muscle cell physiology and contributes to muscle wasting. For instance, decreased miR-29 levels suppress myogenesis and promote muscle atrophy by increasing the expression of atrophy-related genes like MuRF1 and atrogin-1 [83]. Similarly, reduced miR-26a expression is associated with enhanced phosphatase and tensin homolog levels, a negative regulator of the Akt signaling pathway, leading to muscle atrophy [84]. Besides, miRNAs such as miR-23a, miR-27a, and miR-486 improve muscle mass and function by activating Akt and FOXO while blocking SMAD signaling [85,86,87]. However, these miRNAs are downregulated in CKD, increasing phosphatase and tensin homolog and Forkhead box protein O1 (FOXO1) expression, promoting catabolic responses and muscle wasting [85]. These miRNAs also interact with inflammatory pathways, exacerbating muscle degradation. Thus, miRNAs play a critical role in the progression of sarcopenia in CKD by modulating key molecular pathways involved in muscle health. Addressing miRNA dysregulation through targeted therapies could help mitigate sarcopenia in CKD patients. Additionally, CKD enhances nucleolar demethylase expression, reducing ribosomal synthesis and protein translation linking epigenetic changes to sarcopenia [88].

## 6. Specific Therapeutic Approaches for Sarcopenia in CKD

**Figure 3:**
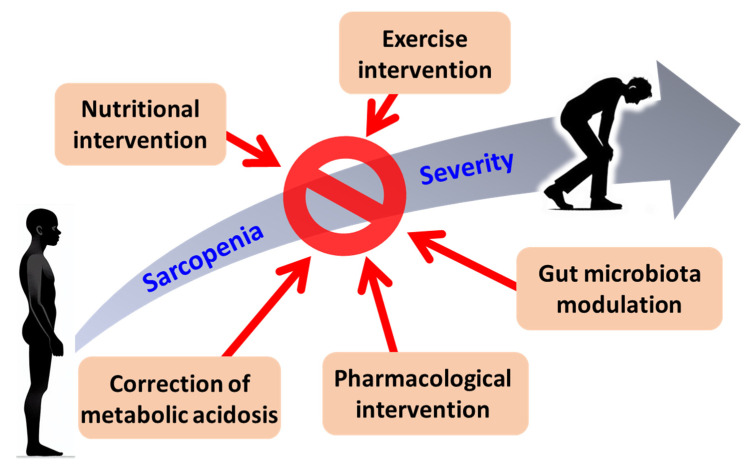
Therapeutic approaches for sarcopenia in CKD.

Current literature has found that sarcopenia could be mitigated by implementing nutritional interventions, exercise interventions, anabolic agents, vitamin D supplementation, correction of metabolic acidosis, gut microbiota modulation, and pharmacological interventions (Figure 3) [89].

### 6.1. Nutritional Interventions

Proper nutrition is vital for preventing and treating sarcopenia in CKD. Nutritional interventions focus on optimizing protein and energy intake to support muscle maintenance and growth. Studies suggest that protein supplementation, particularly with high-quality proteins like whey, can significantly improve muscle mass and function in CKD patients [90]. Essential amino acids, especially leucine, stimulate muscle protein synthesis [91]. The anti-inflammatory effects of omega-3 fatty acids might help reduce muscle degradation.

Furthermore, a balanced diet rich in fruits, vegetables, and whole grains, akin to the Mediterranean diet, has been associated with better muscle health and reduced inflammation. Combining these nutritional strategies with regular physical activity can enhance their effectiveness, improving muscle mass, strength, and overall physical function in CKD patients. Therefore, individualized nutritional plans should be integrated into the comprehensive care of CKD patients to prevent and treat sarcopenia effectively.

Vitamin D is essential to muscle health by regulating calcium and phosphorus metabolism, promoting muscle protein synthesis, and modulating muscle cell proliferation and differentiation [92]. CKD patients often have vitamin D deficiency due to impaired renal synthesis of active vitamin D, leading to muscle weakness and increased risk of sarcopenia [93]. Recent studies have shown the merit of vitamin D supplementation in improving muscle mass and function in CKD patients. For instance, vitamin D supplementation has enhanced muscle strength and physical performance, reduced inflammation, and improved overall HRQoL [94]. A meta-analysis found that vitamin D supplementation significantly mitigates the risk of falls in CKD older adults and simultaneously increases muscle strength. [95]. Additionally, vitamin D’s anti-inflammatory properties may help mitigate the chronic inflammation commonly seen in CKD, contributing to muscle wasting [96]. The optimal dosage and duration of vitamin D supplementation for managing sarcopenia in CKD patients are still under investigation. However, current knowledge suggests that maintaining serum 25-hydroxyvitamin D levels above 30 ng/mL benefits muscle health. For adults, a daily intake of 800–1000 IU of vitamin D is generally recommended, especially for those with severe deficiency or sarcopenia [97]. Despite its potential benefits, vitamin D supplementation was not utilized due to hypercalcemia and vascular calcification concerns in CKD patients. Therefore, monitoring serum calcium and phosphorus levels regularly and adjusting the dosage accordingly is essential. Overall, when used appropriately, vitamin D supplementation can serve as a meaningful approach to managing sarcopenia in CKD patients.

Combining these nutritional strategies with regular physical activity can enhance their effectiveness, improving muscle mass, strength, and overall physical function in CKD patients. Therefore, individualized nutritional plans should be integrated into the comprehensive care of CKD patients to prevent and treat sarcopenia effectively.

### 6.2. Exercise Interventions

Exercise interventions are also pivotal in managing sarcopenia in CKD patients. Recent research has depicted that the protein synthesis rates of skeletal muscle can be stimulated by physical activity, with post-absorptive muscle protein synthesis rates being elevated for up to 24–48 h; such an effect is more prominent before food intake, and vice versa [98]. Hence, the relationship between sarcopenia and physical activity was found significant, implying a potential treatment for sarcopenia [98].

Specifically, aerobic and resistance exercises are fundamental in managing sarcopenia in CKD patients. Resistance exercise is particularly effective, enhancing muscle mass, strength, and physical performance [99]. High-intensity resistance training (80% of one-repetition maximum) is recommended to achieve maximal strength gains [99]. Additionally, aerobic exercises like walking and cycling improve cardiovascular health and overall physical function [99]. Combining resistance and aerobic exercises in a multimodal approach can provide comprehensive benefits, addressing both muscle and cardiovascular health [100].

The recently published Italian Society of Nephrology’s consensus statement [101] highlights that regular physical activity and exercise training improve physical function, cardiometabolic and neuromuscular health, cognitive function, and overall HRQoL in CKD patients. These benefits are evident across all CKD stages, patients receiving dialysis intervention, and kidney transplant recipients. Exercise also offers nephroprotection through glucose tolerance control, body weight regulation, and blood pressure stabilization [101]. Despite its advantages, exercise is underutilized in clinical practice due to the barriers patients and healthcare staff face, such as patient fatigue, lack of motivation, and limited access to exercise facilities. Integrating exercise into routine care plans and using a multidisciplinary team approach can significantly improve outcomes for CKD patients with sarcopenia [101].

### 6.3. Correction of Metabolic Acidosis

Metabolic acidosis, a common complication in CKD, contributes to muscle protein breakdown. Correcting this condition through bicarbonate supplementation can improve protein metabolism, increase serum albumin levels, and enhance muscle mass. This intervention also helps improve overall nutritional status and physical performance.

A randomized controlled trial (RCT) with 188 CKD patients assessed the impact of correcting metabolic acidosis on body composition and kidney function. The intervention group received standard care plus sodium bicarbonate, while the control group received standard care alone. The intervention group was found to have higher lean body mass, MAMC, and estimated glomerular filtration rate. The study concluded that alkali supplementation to increase venous bicarbonate levels to 24–26 mEq/L is associated with preserving lean body mass and kidney function in patients with CKD stages 3 and 4 [102].

The findings are bolstered by recent research by Yang TY et al. [103], which enrolled 14 RCTs comprising 2037 CKD patients with metabolic acidosis. The study demonstrated that sodium bicarbonate supplementation is associated with significantly increased MAMC compared with those without (standardized mean difference, 0.23; 95% confidence interval, 0.08 to 0.38; *p* = 0.003, I2 < 0.001) and concluded the potential benefits of sodium bicarbonate supplementation in increasing muscle mass.

### 6.4. Gut Microbiota Modulation

Research has revealed that the role of gut microbiota dysbiosis in CKD-associated sarcopenia is significant [43]. Modulating the gut microbiota through probiotics, prebiotics, and synbiotics can help reduce uremic toxins and inflammation, improving muscle health. AST-120, an oral adsorbent, has shown promise in lowering indoxyl sulfate and p-cresyl sulfate levels, ameliorating muscle atrophy, and enhancing exercise capacity [104]. Additionally, emerging therapies targeting the gut–muscle axis, such as probiotics and prebiotics, hold promise in modulating gut microbiota and reducing uremic toxin levels. These interventions could further support muscle health in CKD patients.

### 6.5. Pharmacological Interventions

Pharmacological interventions are emerging as promising therapeutic approaches for managing sarcopenia in CKD patients. Experiments with animals have observed protein synthesis through the inactivation of FOXO and mTOR in insulin or insulin-like growth factor (IGF)-1-phosphatidylinositol 3-kinase-Akt pathway [33,105,106,107,108]. Drugs relative to the mechanisms of protein synthesis include anabolic hormones, myostatin inhibitors, selective androgen receptor modulators, and mTOR inhibitors, which help stimulate muscle protein synthesis and reduce muscle degradation. Anabolic hormones increase muscle mass and strength, including testosterone, growth hormone, and IGF-1 [109]. Myostatin inhibitors, which block the myostatin pathway that negatively regulates muscle growth, are also being explored for their potential to enhance muscle hypertrophy and function [110].

Another promising area is the use of anti-inflammatory agents to reduce chronic inflammation, a key contributor to muscle wasting in CKD. For instance, omega-3 fatty acids were found to mitigate muscle degradation by possessing anti-inflammatory properties [111]. Furthermore, AST-120 has demonstrated potential in reducing uremic toxins, which are implicated in muscle atrophy and decreased exercise capacity [104]. Meanwhile, anorexia is common in CKD patients, which consists of complex interplays between metabolic signals and anomalies in the organ systems, as well as an iterative dominance process of physical and psychological [112]. Gastric mediators, cytokines, and adipokines mediate anorexia [113]. Hence, megestrol acetate, an appetite stimulant, has also increased weight, protein catabolic rate, and muscle mass.

## 7. Future Perspectives

Future perspectives on sarcopenia in CKD involve a multifaceted approach to better understand and manage this condition. Key areas include elucidating molecular pathways leading to muscle wasting, focusing on inflammation, hormonal imbalances, and metabolic disturbances [5]. Advanced omics technologies, such as genomics and proteomics, are expected to provide deeper insights and identify novel therapeutic targets. Exploring the gut–muscle axis is promising, as gut microbiota dysbiosis contributes to muscle wasting. Modulating the gut microbiota through probiotics and prebiotics could offer new therapeutic avenues.

Additionally, understanding epigenetic modifications influencing muscle health could lead to innovative treatments. Personalized medicine approaches are crucial, tailoring interventions based on genetic, metabolic, and lifestyle factors. This includes personalized dietary plans, exercise regimens, and pharmacological therapies. Digital health technologies like wearable devices and telemedicine can facilitate continuous monitoring and personalized adjustments. Ongoing clinical trials are essential to validate new interventions. Collaboration among researchers, clinicians, and patients is critical to translate findings into practice. Also, tackling challenges related to exercise and nutritional interventions is necessary for successful implementation. In summary, future research should focus on understanding sarcopenia mechanisms in CKD, exploring novel therapeutic targets, and developing personalized, multidisciplinary approaches to improve patient outcomes [85,114,115,116,117,118].

## 8. Conclusions

Sarcopenia in CKD is a multifaceted condition influenced by metabolic, hormonal, inflammatory, and nutritional factors. This review identifies factors associated with sarcopenia in CKD and the complex interplay between these mechanisms and emphasizes comprehensive management. Nutritional interventions such as adequate protein, vitamin D supplementation, and regular physical activity are essential for mitigating muscle wasting. Pharmacological approaches with anabolic agents and anti-inflammatory treatments show promise, but they will need testing on lots of other patients to establish their best regimen. Newer modalities include those targeting the gut–muscle axis and epigenetic modifications. Future research should focus on elucidating the precise molecular pathways involved in sarcopenia, identifying novel biomarkers for early detection, and developing personalized, multidisciplinary treatment strategies. Enhancing our understanding and managing sarcopenia in CKD can improve patient outcomes and quality of life.

## Figures and Tables

**Table 1:** Summary of the contents of EWGSOP2 and AWGS.

	EWGSOP2	AWGS
Measurement		
Case findings for further sarcopenia evaluation	- Clinical suspicion or SARC-F ≥ 4 [13]	- With any of the following: clinical conditions (e.g., functional decline, unintentional weight loss, depressive mood, etc.) or comorbidities (e.g., heart failure, COPD, DM, CKD, etc.) - Calf circumference (<34 cm for men, <33 cm for women) or SARC-F ≥ 4, or SARC-F/Calf ≥ 11 - DXA < 7.0 kg/m^2^ (M), <5.4 kg/m^2^ (F) - Bioimpedance < 7.0 kg/m^2^ (M), <5.7 kg/m^2^ (F)
Cut-off points		
Muscle strength	- HGS: <27 kg (M), <16 kg (F) or 5 times chair stand test: >15 s	- HGS: <28 kg (M), <18 kg (F)
Muscle quantity or quality	- ASM: <20 kg (M), <15 kg (F) or - ASMI: <7.0 kg/m^2^ (M), <5.5 kg/m^2^ (F)	- ASM: <7.0 kg/m^2^ (M), or <5.4 kg/m^2^ (F, by DXA), or <5.7 kg/m^2^ (F, by BIA)
Physical performance	- Gait speed ≤ 0.8 m/s - SPPB: ≤8 points - TUG: ≥20 s	- 6MWT < 1.0 m/s or - SPPB ≤ 9 points or - 5-time Chair stand test ≥ 12 s
Classification		
Probable sarcopenia	Low muscle strength	N/A
Confirmed sarcopenia	Low muscle strength + Low muscle quantity or quality	Low ASM + low muscle strength or low physical performance
Severe sarcopenia	Low muscle strength + low muscle quantity or quality + low Physical performance	Low ASM + low muscle strength or low physical performance

Abbreviations: ASM, Appendicular Skeletal Muscle Mass; ASMI, ASM/height squared; AWGS, Asian Working Group for Sarcopenia; CKD, Chronic kidney disease; COPD, Chronic obstructive pulmonary disease; DM, Diabetes mellitus; DXA, Dual Energy X-Ray Absorptiometry; EWGSOP2, European Working Group on Sarcopenia in Older People; HGS, handgrip strength; SARC-f, Sarcopenia Assessment Tool Questionnaire; SARC-F/Calf, SARC-F Questionnaire with Calf Circumference; SPPB, Short Physical Performance Batter; TUG, Timed up and Go test; 6MWT, 6 min walking test. Note: F denotes female, M denotes male.

## Data Availability

Not applicable.
